# Models of disease behavior in idiopathic pulmonary fibrosis

**DOI:** 10.1186/s12916-015-0403-7

**Published:** 2015-09-24

**Authors:** Kerri A. Johannson, Brett Ley, Harold R. Collard

**Affiliations:** Department of Medicine, University of Calgary, 6th Floor - 4448 Front Street S.E., Calgary, AB T3M-1M4 Canada; Department of Medicine, University of California, San Francisco, Box 0111, 505 Parnassus Avenue, San Francisco, CA 94143 USA

**Keywords:** Biological markers, Idiopathic pulmonary fibrosis, Interstitial lung disease, Prognosis

## Abstract

Idiopathic pulmonary fibrosis is a diffuse parenchymal lung disease of unknown cause. The natural history of disease can vary considerably, making it difficult to predict the clinical trajectory for an individual patient. Accurate prognostication is desirable for clinical management as well as for cohort enrichment in clinical trials of therapeutics. Clinical and biomarker models of disease behavior have been developed to improve prognostication in idiopathic pulmonary fibrosis, with moderate predictive capabilities. Integrated prediction models that combine both clinical and biomarker variables will improve prognostication for patients and improved cohort enrichment strategies for clinical trials. This goal may be best achieved through collaborative patient registries with prospectively collected biological samples that allow for characterization of disease behavior in idiopathic pulmonary fibrosis.

## Background

Idiopathic pulmonary fibrosis (IPF) is a diffuse parenchymal lung disease of unknown etiology associated with a median survival of 3 to 5 years after diagnosis [1]. Disease behavior is variable among patients, with some individuals remaining relatively stable over long periods, while others may experience a slow progressive decline, rapid decline, or suffer acute exacerbation [2]. Predicting the clinical course in IPF is challenging due to the heterogeneous nature of the disease, but it remains a critically important goal for both clinical and research purposes. Knowledge of an individual’s probability of disease progression or risk of death may affect timing of drug therapies or listing for lung transplantation. In clinical trials of therapeutics, accurate prognostication is desirable to maximize the likelihood of detecting treatment effects through cohort enrichment. For these reasons, several models of disease behavior for IPF have been developed with the common goal of accurate prognostication. Each model has contributed valuably to our understanding of IPF, identifying key clinical, physiologic, radiologic, pathologic, and biologic features associated with outcomes of interest.

## Clinical models of disease behavior

Early risk prediction models incorporated baseline clinical and radiographic parameters to predict mortality in IPF. The composite clinical, radiological, and physiological scoring system identified age, clubbing, smoking history, lung volumes, end-exercise hypoxemia, and chest radiographic evidence of pulmonary hypertension and interstitial abnormalities to be associated with survival [3, 4]. The Composite Physiologic Index was similarly developed, incorporating three lung function parameters to predict mortality and accounting for the confounding effects of concomitant emphysema in IPF patients [5], a limitation of prior models.

More recently, du Bois et al. [6] developed a risk scoring system based on age, history of respiratory hospitalization, baseline forced vital capacity (FVC), and change in FVC over 24 weeks to predict mortality. This was subsequently modified to include a functional and longitudinal parameter, the 6-minute walk distance (6MWD) and change in this parameter over 24 weeks [7]. Ley et al. [8] derived and validated the ‘gender, age, physiology’ (GAP) model, which identified four readily available baseline parameters, namely gender, age, FVC, and diffusion capacity of the lung for carbon monoxide (D_LCO_), to develop staging and risk prediction scores. An alternative model in which the extent of fibrosis on high resolution computed tomography of the chest was used in place of the D_LCO_ performed equally well [9]. The original du Bois and GAP models have subsequently been combined to provide an integrated baseline and longitudinal risk prediction approach [10].

These clinical models have demonstrated the impact of cohort characteristics on calibration of risk. This is most obvious in comparing risk in referral center-based cohorts and clinical trial cohorts. Models derived in center-based cohorts appear to significantly overestimate mortality risk in clinical trial cohorts, where patients are highly selected [11]. Additionally, age and gender appear to be more relevant prognostic variables in clinical cohorts, perhaps through capturing the influence of comorbidities, while adding relatively little in a clinical trial cohort. Thus, calibration of risk prediction models to the population of interest appears critical to accurate quantification of risk.

Clinical risk prediction models provide important prognostic tools for practice and clinical trial development. However, their performance remains modest, likely because clinical markers are limited in their inability to directly assess the underlying pathobiology and disease activity. Translational studies are providing novel tools in the form of molecular and genetic biomarkers to address this limitation.

## Molecular and genetic biomarker-based models of disease behavior

Several recent studies have identified molecular and genetic biomarkers associated with clinical outcomes in IPF [12]. These can be divided into three categories: genetic, protein, and cellular.

Genetic-based biomarkers associated with worse survival in IPF include mucin 5B promoter polymorphisms [13], shorter leucocyte telomere length [14], and the toll-interacting protein single nucleotide polymorphism [15]. Protein-based biomarkers that have been associated with worse outcomes in IPF include surfactant proteins A (SP-A) [16] and D [17], Krebs von den Lungen-6 (KL-6) [18, 19], CC-chemokine-ligand-18 [20], C-X-C motif chemokine 13 [21, 22], matrix metalloproteinase (MMP)-3 [22] and MMP-7 [23], fibulin-1 [24], interleukin-8 and intercellular cell adhesion molecule-1 [23], osteopontin [25], periostin [26, 27], and collagen degradation products [28]. Cellular biomarkers associated with worse outcomes in IPF include regulatory T cells (Tregs) [29], semaphorin 7a + Tregs [30], and circulating fibrocytes [31].

Molecular and genetic biomarkers seem certain to add to the predictive abilities of currently available clinical risk prediction models. To date, few studies have examined this additive benefit and rigorous validation is lacking, but superior model performance has been suggested with certain combinations of clinical variables and biomarkers [13, 18, 23, 32]. Song et al. [18] proposed that the combination of at least three biomarkers (e.g., MMP-7, SP-A, and KL-6) improved risk prediction over clinical variables alone. Clearly, more needs to be done to clarify the additive role of molecular and genetic biomarkers.

## Conclusions

Taken together, these early reports highlight the potential for more accurate modeling of disease behavior in IPF. However, several important limitations remain. First, while survival is unquestionably a clinically meaningful outcome, it is of less use to patients and clinicians than pre-mortality outcomes such as disease progression. No model to date accurately predicts pre-mortality outcomes such as loss of lung function or acute exacerbation. Second, available models demonstrate only modest prediction accuracy. Potential explanations for this include the inability to capture other co-morbidities (e.g., cardiac disease, cancer) leading to death in IPF patients, the lack of reliable biomarkers of disease activity, and the failure to account for processes such as acute exacerbation. Lastly, quantification of risk may differ between patient populations, suggesting that models may need to be tailored to the population of interest.

Future research will need to address these and other limitations. We anticipate that models that combine clinical and biological variables will lead to improved prognostication for patients and improved cohort enrichment strategies in clinical trials. To develop these integrated models, we believe that a centralized registry of well-characterized patients with systematically collected bio-specimens will prove essential [33].

## Abbreviations

D_LCO_: Diffusion capacity of the lung for carbon monoxide
FVC: Forced vital capacity
IPF: Idiopathic pulmonary fibrosis

## References

[1] Raghu G, Collard HR, Egan JJ, Martinez FJ, Behr J, Brown KK (2011). An official ATS/ERS/JRS/ALAT statement: idiopathic pulmonary fibrosis: evidence-based guidelines for diagnosis and management. Am J Respir Crit Care Med.

[2] Collard HR, Moore BB, Flaherty KR, Brown KK, Kaner RJ, King TE (2007). Acute exacerbations of idiopathic pulmonary fibrosis. Am J Respir Crit Care Med.

[3] King TE, Tooze JA, Schwarz MI, Brown KR, Cherniack RM (2001). Predicting survival in idiopathic pulmonary fibrosis: scoring system and survival model. Am J Respir Crit Care Med.

[4] Watters LC, King TE, Schwarz MI, Waldron JA, Stanford RE, Cherniack RM (1986). A clinical, radiographic, and physiologic scoring system for the longitudinal assessment of patients with idiopathic pulmonary fibrosis. Am Rev Respir Dis.

[5] Wells AU, Desai SR, Rubens MB, Goh NS, Cramer D, Nicholson AG (2003). Idiopathic pulmonary fibrosis: a composite physiologic index derived from disease extent observed by computed tomography. Am J Respir Crit Care Med.

[6] du Bois RM, Weycker D, Albera C, Bradford WZ, Costabel U, Kartashov A (2011). Ascertainment of individual risk of mortality for patients with idiopathic pulmonary fibrosis. Am J Respir Crit Care Med.

[7] du Bois RM, Albera C, Bradford WZ, Costabel U, Leff JA, Noble PW (2014). 6-Minute walk distance is an independent predictor of mortality in patients with idiopathic pulmonary fibrosis. Eur Respir J.

[8] Ley B, Ryerson CJ, Vittinghoff E, Ryu JH, Tomassetti S, Lee JS (2012). A multidimensional index and staging system for idiopathic pulmonary fibrosis. Ann Intern Med.

[9] Ley B, Elicker BM, Hartman TE, Ryerson CJ, Vittinghoff E, Ryu JH (2014). Idiopathic pulmonary fibrosis: CT and risk of death. Radiology.

[10] Ley B, Bradford WZ, Weycker D, Vittinghoff E, du Bois RM, Collard HR (2015). Unified baseline and longitudinal mortality prediction in idiopathic pulmonary fibrosis. Eur Respir J.

[11] Collard HR, Brown KK, Martinez FJ, Raghu G, Roberts RS, Anstrom KJ (2014). Study design implications of death and hospitalization as end points in idiopathic pulmonary fibrosis. Chest.

[12] Ley B, Brown KK, Collard HR (2014). Molecular biomarkers in idiopathic pulmonary fibrosis. Am J Physiol Lung Cell Mol Physiol.

[13] Peljto AL, Zhang Y, Fingerlin TE, Ma SF, Garcia JG, Richards TJ (2013). Association between the MUC5B promoter polymorphism and survival in patients with idiopathic pulmonary fibrosis. JAMA.

[14] Stuart BD, Lee JS, Kozlitina J, Noth I, Devine MS, Glazer CS (2014). Effect of telomere length on survival in patients with idiopathic pulmonary fibrosis: an observational cohort study with independent validation. Lancet Respir Med.

[15] Noth I, Zhang Y, Ma SF, Flores C, Barber M, Huang Y (2013). Genetic variants associated with idiopathic pulmonary fibrosis susceptibility and mortality: a genome-wide association study. Lancet Respir Med.

[16] Greene KE, King TE, Kuroki Y, Bucher-Bartelson B, Hunninghake GW, Newman LS (2002). Serum surfactant proteins-A and -D as biomarkers in idiopathic pulmonary fibrosis. Eur Respir J.

[17] Barlo NP, van Moorsel CH, Ruven HJ, Zanen P, van den Bosch JM, Grutters JC (2009). Surfactant protein-D predicts survival in patients with idiopathic pulmonary fibrosis. Sarcoidosis Vasc Diffuse Lung Dis.

[18] Song JW, Do KH, Jang SJ, Colby TV, Han S, Kim DS (2013). Blood biomarkers MMP-7 and SP-A: predictors of outcome in idiopathic pulmonary fibrosis. Chest.

[19] Yokoyama A, Kohno N, Hamada H, Sakatani M, Ueda E, Kondo K (1998). Circulating KL-6 predicts the outcome of rapidly progressive idiopathic pulmonary fibrosis. Am J Respir Crit Care Med.

[20] Prasse A, Probst C, Bargagli E, Zissel G, Toews GB, Flaherty KR (2009). Serum CC-chemokine ligand 18 concentration predicts outcome in idiopathic pulmonary fibrosis. Am J Respir Crit Care Med.

[21] Vuga LJ, Tedrow JR, Pandit KV, Tan J, Kass DJ, Xue J (2014). C-X-C motif chemokine 13 (CXCL13) is a prognostic biomarker of idiopathic pulmonary fibrosis. Am J Respir Crit Care Med.

[22] DePianto DJ, Chandriani S, Abbas AR, Jia G, N’Diaye EN, Caplazi P (2015). Heterogeneous gene expression signatures correspond to distinct lung pathologies and biomarkers of disease severity in idiopathic pulmonary fibrosis. Thorax.

[23] Richards TJ, Kaminski N, Baribaud F, Flavin S, Brodmerkel C, Horowitz D (2012). Peripheral blood proteins predict mortality in idiopathic pulmonary fibrosis. Am J Respir Crit Care Med.

[24] Jaffar J, Unger S, Corte TJ, Keller M, Wolters PJ, Richeldi L (2014). Fibulin-1 predicts disease progression in patients with idiopathic pulmonary fibrosis. Chest.

[25] Kadota J, Mizunoe S, Mito K, Mukae H, Yoshioka S, Kawakami K (2005). High plasma concentrations of osteopontin in patients with interstitial pneumonia. Respir Med.

[26] Naik PK, Bozyk PD, Bentley JK, Popova AP, Birch CM, Wilke CA (2012). Periostin promotes fibrosis and predicts progression in patients with idiopathic pulmonary fibrosis. Am J Physiol Lung Cell Mol Physiol.

[27] Okamoto M, Hoshino T, Kitasato Y, Sakazaki Y, Kawayama T, Fujimoto K (2011). Periostin, a matrix protein, is a novel biomarker for idiopathic interstitial pneumonias. Eur Respir J.

[28] Jenkins RG, Simpson JK, Saini G, Bentley JH, Russell AM, Braybrooke R (2015). Longitudinal change in collagen degradation biomarkers in idiopathic pulmonary fibrosis: an analysis from the prospective, multicentre PROFILE study. Lancet Respir Med.

[29] Kotsianidis I, Nakou E, Bouchliou I, Tzouvelekis A, Spanoudakis E, Steiropoulos P (2009). Global impairment of CD4 + CD25 + FOXP3+ regulatory T cells in idiopathic pulmonary fibrosis. Am J Respir Crit Care Med.

[30] Reilkoff RA, Peng H, Murray LA, Peng X, Russell T, Montgomery R (2013). Semaphorin 7a + regulatory T cells are associated with progressive idiopathic pulmonary fibrosis and are implicated in transforming growth factor-beta1-induced pulmonary fibrosis. Am J Respir Crit Care Med.

[31] Moeller A, Gilpin SE, Ask K, Cox G, Cook D, Gauldie J (2009). Circulating fibrocytes are an indicator of poor prognosis in idiopathic pulmonary fibrosis. Am J Respir Crit Care Med.

[32] Kinder BW, Brown KK, McCormack FX, Ix JH, Kervitsky A, Schwarz MI (2009). Serum surfactant protein-A is a strong predictor of early mortality in idiopathic pulmonary fibrosis. Chest.

[33] White ES, Brown KK, Collard HR, Conoscenti CS, Cosgrove GP, Flaherty KR (2014). Open-access biorepository for idiopathic pulmonary fibrosis. The way forward. Ann Am Thorac Soc.

